# Acute Disseminated Encephalomyelitis: Current Perspectives

**DOI:** 10.3390/children7110210

**Published:** 2020-11-03

**Authors:** Renata Barbosa Paolilo, Kumaran Deiva, Rinze Neuteboom, Kevin Rostásy, Ming Lim

**Affiliations:** 1 Department of Neurology, Hospital das Clínicas, Faculty of Medicine, University of São Paulo (HCFMUSP), São Paulo 05508-060, Brazil; renatabpaolilo@gmail.com; 2 Department of Pediatric Neurology, Assistance Publique-Hôpitaux de Paris, University Hospitals Paris Saclay, Bicêtre Hospital, 72, Rue G Leclerc, 94270 Le Kremlin Bicêtre, France; Kumaran.deiva@aphp.fr; 3 National Reference Centre for Rare Inflammatory Brain and Spinal Diseases, 72, Rue G Leclerc, 94270 Le Kremlin Bicêtre, France; 4 Inserm UMR 1184, Immunology of Viral Infections and Autoimmune Diseases, 63, R G Péri, 94270 Le Kremlin Bicêtre, France; 5 Department of Neurology, Erasmus University Medical Center, 3015 GD Rotterdam, The Netherlands; r.neuteboom@erasmusmc.nl; 6 Department of Pediatric Neurology, Vestische Kinder und Jugendklinik, Witten/Herdecke University, 45711 Datteln, Germany; k.rostasy@kinderklinik-datteln.de; 7 Children’s Neurosciences, Evelina London Children’s Hospital at Guy’s and St Thomas’ NHS Foundation Trust, London SE1 7EH, UK; 8 King’s Health Partners Academic Health Science Centre, London SE1 9RT, UK; 9 Faculty of Life Sciences and Medicine, King’s College Hospital, London SE5 9RS, UK

**Keywords:** acute disseminated encephalomyelitis (ADEM), acquired demyelination syndrome (ADS), myelin oligodendrocyte glycoprotein antibody (MOG-Ab), multiple sclerosis (MS), optic neuritis (ON), neuromyelitis optica spectrum disorder (NMOSD)

## Abstract

Acute disseminated encephalomyelitis (ADEM) is an immune-mediated central nervous system (CNS) disorder, characterized by polyfocal symptoms, encephalopathy and typical magnetic resonance imaging (MRI) findings, that especially affects young children. Advances in understanding CNS neuroimmune disorders as well as the association of myelin oligodendrocyte glycoprotein antibody (MOG-Ab) with both monophasic and recurrent forms of ADEM have led to new insights into its definition, management and outcome. In this review, we aim to provide an update based on current epidemiologic, clinical, radiological and immunopathological aspects and clinical outcome of ADEM.

## 1. Brief Introduction and Historical Perspective

Acute disseminated encephalomyelitis (ADEM) is an inflammatory demyelinating disorder of the central nervous system (CNS) that predominantly affects children [1]. Whilst the first ADEM descriptions date back to the 18th century, the understanding of its physiopathology and definition have continued to evolve [2]. In the last decade, in an effort to characterize the range of acute demyelinating syndromes, the International Pediatric Multiple Sclerosis Study Group (IPMSSG) produced consensus clinical and radiologic diagnostic criteria defining ADEM as an acute demyelination syndrome that presents clinically with encephalopathy and polyfocal CNS symptoms showing demyelination on brain magnetic resonance imaging (MRI) [3,4]. As the imaging criteria were predicated on not having features characteristic of multiple sclerosis, challenges have arisen when utilizing imaging to diagnose ADEM [5] and predict other relapsing syndromes [6,7]. A key development in recent years is the recognition of the association of myelin oligodendrocyte glycoprotein antibody (MOG-Ab) with ADEM and recurrent forms of demyelination such as multiphasic ADEM (MDEM) and ADEM followed by recurrent optic neuritis (ADEM-ON) [1,8,9]. Insights into MOG-Ab-associated disease manifestation are starting to challenge older assumptions of ADEM [10,11,12]. Despite these advances, little change in acute therapeutic strategies has occurred, although the newer paradigm of timely initiation and optimization of immune treatments employed for a range of neuroinflammatory conditions is also beginning to be utilized for demyelinating disorders [13]. Protocols focus on early high-dose intravenous corticosteroids as first-line therapy and immunoglobulin and plasma exchange as second-line therapy with overall good outcomes based on observational studies [1,10]. The timing of treatment escalation and re-assessment of radiological findings remain unsolved questions. As for outcomes, which have historically been considered favorable, consistent findings have yet to shed light on the cognitive impairment reported in a few cohorts [1,14,15]. Here, we target this review on the clinical, epidemiological and pathophysiological aspects of pediatric ADEM, focusing on newer clinical perspectives.

## 2. ADEM in the Context of ADS

**Figure 1:**
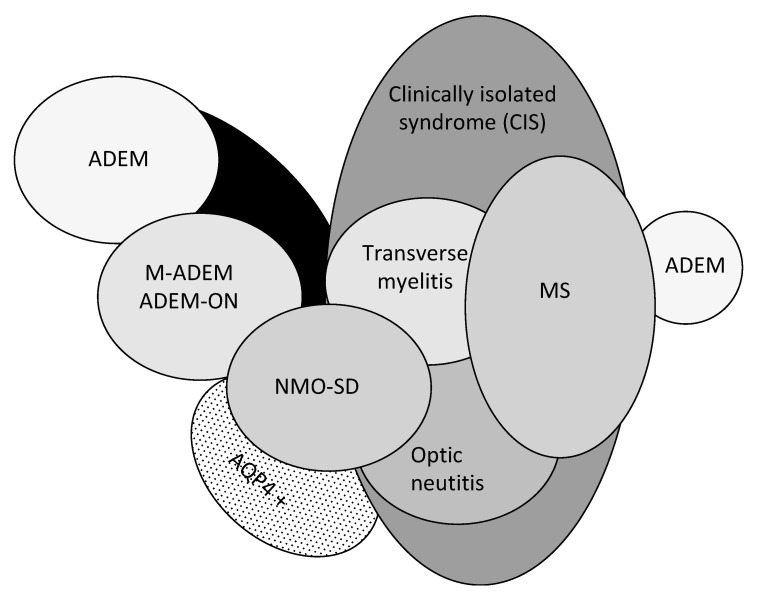
Spectrum of acquired demyelinating syndromes. ADEM, acute disseminated encephalomyelitis; MOG, myelin oligodendrocyte glycoprotein; AQP4 +, aquaporin-4; M-ADEM, multiphasic-ADEM; ADEM-ON, ADEM followed by at least one optic neuritis; NMOSD, neuromyelitis optica spectrum disorder; MS, multiple sclerosis (adapted from Neuteboom et al. [16]).

Pediatric ADEM belongs to a group of disorders (Figure 1) characterized by acute or subacute onset of neurological deficits associated with evidence of inflammatory demyelination of the CNS, including the optic nerves, collectively named acquired demyelinating syndromes (ADSs) [4,16,17]. ADEM is a relatively common subtype of ADS, occurring in 22–32% of children with ADS [12,18,19,20,21].

Direct comparisons among earlier cohorts have been difficult because of varying definitions, as highlighted by Mikaeloff and Tardieu [22]. As such, differences across a range of many other demyelinating conditions catalyzed an international effort by the IPMSSG to develop diagnostic criteria [3,4]. The distinct subtypes of ADS are typically classified based on (1) clinical localization of symptoms and signs and (2) whether they are monophasic or relapsing diseases.

The first event of ADS in children remains monophasic in most cases and is mainly classified as ADEM or clinically isolated syndrome (CIS). By definition, ADEM requires the presence of encephalopathy and polyfocal CNS symptoms. As for CIS, the definition is applied for ADS presentations that can be either monofocal or polyfocal, reflecting clinical signs and symptoms in one or multiple CNS locations. Typical monofocal presentations are optic neuritis (ON), brainstem symptoms (i.e., internuclear ophthalmoplegia) and transverse myelitis (TM).

The first demyelinating event might also represent the first manifestation of a chronic demyelinating relapsing disease, such as multiple sclerosis (MS), neuromyelitis optica spectrum disorder (NMOSD) or myelin oligodendrocyte glycoprotein antibody (MOG-Ab)-associated disease, recently recognized as a multiphasic disease course that does not resemble MS [23,24]. ADEM as a first manifestation of MS or NMOSD is uncommon <10% [1,25]. Typical brain MRI characteristics and aquaporin-4 antibody (AQP4-Ab), respectively, help to distinguish these disorders [9]. This process of diagnostic refinement is still ongoing, illustrating the need for further biomarkers.

The IPMSSG definitions for monophasic ADEM and relapsing disorders are shown in Table 1.

## 3. Epidemiology

Although it can afflict people at any age, ADEM is more common in children, with a median age at presentation of 5–8 years [26]. A male preponderance had been shown in most studies, with a male:female ratio ranging from 1:0.8 to 2.3:1 [27].

Reported annual incidence varies from 0.07–0.9/100,000 children in different locations [1,7,17,27,28,29,30]. It has been described throughout the world and affects all ethnicities, but its incidence is higher with increasing distance from the Equator, similar to the geographic distribution of MS [31].

Mechanistically, ADEM has been classified as a predominately post-infectious CNS disorder, with an identifiable trigger reported in up to 50–85% of cases [28,32]. First ADEM descriptions were commonly associated with infections such as smallpox and measles, with high mortality and neurological sequelae. Although immunization programs reduced the incidence of ADEM related to those infections, ADEM continues to be associated with other infections, especially, but not exclusively, viral infections. Neurological symptoms generally begin within 2–21 days (range 1–42) after an infection, and the most frequent are flu-like symptoms, upper respiratory tract infection and gastroenteritis. Bacterial infections have also been implicated [26,28]. Although some studies [32] required previous infection for ADEM diagnosis, this was not included in the current diagnostic criteria.

Although vaccinations have also been implicated as a cause of ADEM, no clear pathogenetic correlation exists and the incidence of ADS following infection is higher than that induced by immunization itself [1,33,34]. The frequency of ADEM occurring after vaccination has decreased in recent years, probably due to changes in the methods used to produce vaccines [33]. Almost all immunizations have been implicated, especially rabies, hepatitis B, polio, influenza, pertussis, measles, mumps and rubella [26]. A first vaccination is more associated with ADEM than re-vaccination [33]. A recent large case–control study found no association of vaccines with ADS in a three-year follow-up period of both children and adults [34]. This finding emphasizes that vaccines might have a similar effect as infections in triggering the first episode of ADS and may accelerate the transition from subclinical to relapsing ADS in patients with an existing risk for autoimmune disease.

## 4. Clinical and Radiological Perspective

### 4.1. Diagnostic Criteria

The diagnosis of ADEM is based on a combination of clinical features, supported by MRI findings (see Table 1) [4]. Importantly, other diseases need to be excluded before a definite diagnosis can be made. Based on the most recent IPMSSG criteria, ADEM is characterized by polyfocal neurological deficits. Notably, the presence of encephalopathy is an obligatory feature. The definition of encephalopathy encompasses behavioral changes and/or alterations in consciousness, including irritability, not explained by systemic febrile illness or post-ictal symptoms. The obligatory presence of encephalopathy has been debated but remains present in the criteria, mainly due to the lack of sensitive and specific MRI criteria, along with serum or CSF (cerebrospinal fluid) biomarkers [21,35,36]. As there may be fluctuations in the disease course in the first three months, i.e., treatment-related fluctuations caused by cessation or tapering of steroids, any worsening, recurring or new symptoms within three months are still attributed to the first event. If there is a second event after three months that again qualifies as ADEM, the term multiphasic ADEM is used. The term recurrent ADEM, suggested in the 2007 IPMSSG criteria, was omitted in the 2012 IPMSSG criteria due to its low frequency in reported studies [3,4]. Another multiphasic phenotype is ADEM followed by (recurrent) optic neuritis (ADEM-ON). A diagnosis of MS after a first event of ADEM is rare, but possible if a child with ADEM experiences two subsequent non-encephalopathic events or one new event with the appearance of new MS-specific MRI lesions that are dispersed across time and space.

### 4.2. Clinical Features

A preceding infection or illness is observed in 70–80% of cases [37]. Although often preceded by a prodromal phase (malaise, headache), disease onset is subacute to acute. Progression of the disease usually takes places within days. Neurological signs include pyramidal signs, ataxia, brainstem symptoms, optica neuritis and transverse myelitis [1,37,38]. Symptoms may also include atypical signs like meningism, fever and seizures, resembling infectious meningo-encephalitis. Intensive care unit (ICU) management is reported for 15% of children with ADEM [38]. Clinical recovery typically occurs over weeks after the peak. Rarely, systemic involvement can occur, including cardiac complications with myocardial tissue damage [39,40].

### 4.3. MRI Findings

**Figure 2:**
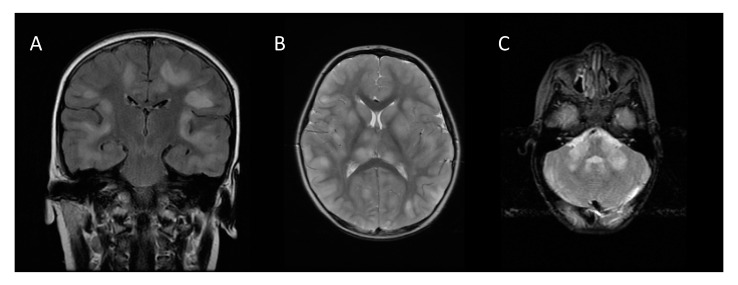
Typical magnetic resonance imaging features of ADEM: (**A**) coronal fluid-attenuated inversion recovery (FLAIR) image showing asymmetrical bilateral subcortical white matter abnormalities; (**B**) axial T2-weighted image depicting asymmetrical bilateral white and gray matter abnormalities; (**C**) axial T2-weighted image showing typical cerebellar peduncle lesions.

Brain MRI in the acute stage shows hyperintense abnormalities in T2-weighted and fluid-attenuated inversion recovery (FLAIR) images [4]. Lesions typically are bilateral, asymmetrical, large (>2 cm) and poorly demarcated. Both white and gray matter can be affected. Cortical as well as deep gray matter lesions have been described. Figure 2 shows some examples of typical ADEM MRI features. Gadolinium enhancement is not a typical feature of ADEM, reported in up to 30% of cases [14]. The frequency of spinal cord abnormalities is not well known, as spinal cord imaging is not routinely done in all ADEM cohort studies. If present, spinal cord abnormalities can be observed over more than two vertebral segments [14,35,38]. Complete resolution of MRI abnormalities has not been studied systematically, likely due to the need for sedation of young children for the procedure. This may be the reason for conflicting data on the resolution of MRI abnormalities [14,41,42].

Notably, MRI may show no abnormalities in the first days [42]. Additionally, during clinical recovery, MRI imaging may still show worsening, indicating a lag between clinical symptoms and MRI abnormalities. As some patients with ADEM may have a multiphasic disease course and even develop MS, a reference scan timed around three months after ADEM onset is advised.

In order to discriminate between ADEM and MS at the time of the first attack, three items are potentially useful: (1) two or more periventricular lesions, (2) presence of black holes and (3) absence of a bilateral lesion pattern. If two of these three items are present, MS is more likely than ADEM. Yet, it should be noted that these criteria are not diagnostic, and the last item especially can be variably interpreted [43,44].

### 4.4. Laboratory Findings

Blood investigations show leucocytosis or elevated C-reactive protein (CRP) or erythrocyte sedimentation rate (ESR) in around 50% of children with ADEM, and therefore do not discriminate between ADEM and infectious meningo-encephalitis [38]. Data on cerebrospinal fluid (CSF) findings differ between studies. CSF pleocytosis is observed in a wide range of patients (28–86%) [14,35,38,45]. There is no strict upper limit to the pleocytosis, although CSF leukocyte cell counts above 100/μL are rarely seen and warrant against the possibility of an infectious meningoencephalitis [38]. Elevated CSF protein levels are observed in 23–66% of cases [14,35,45,46]. This may be an underestimation, as the normal values of CSF proteins in children are lower than in adults [46]. Oligoclonal bands are found in less than 10% and may be transitory, contrary to MS [14,35,38,45].

### 4.5. Differential Diagnosis

The differential diagnosis of ADEM is broad and contains, besides other ADSs, other inflammatory diseases and vascular, metabolic and genetic disorders [1,47]. Stroke-like events should point towards CNS vasculitis, systemic lupus erythematosus (SLE), Behcet’s disease, antiphospholipid antibody syndrome or mitochondrial encephalomyopathy, lactic acidosis and stroke-like episodes (MELASs). Persistent seizures or predominant extrapyramidal movement disorders like dystonia and chorea may be symptoms of anti-N-methyl-D-asparate receptor (anti-NMDAR) encephalitis. This situation generally shows normal MRI, although white matter involvement has been described [13,47]. Bilateral symmetrical lesion patterns should guide the clinician towards diseases such as primary or secondary hemophagocytic lympho-histiocytosis and/or genetic/metabolic disorders and leukodystrophies. A progressive disease course is another red flag and is indicative of genetic/metabolic disorders or neoplastic diseases like gliomatosis cerebri or lymphoma.

### 4.6. Comparison between Children and Adults

Making comparisons between children and adults with ADEM is difficult, as there are no international definitions of ADEM in adults. Although the clinical presentation in children and adults is comparable, there is evidence that the disease course and outcomes are worse in adults than in children [38]. Adults have a higher frequency of ICU admission, longer hospitalization, poorer recovery and higher mortality [38]. In children, MRI abnormalities more often show thalamic and basal ganglia lesions, whereas adults more often show periventricular lesions [35,38] and higher Gad–enhancing lesions (30% vs. 55–60%). A multiphasic, non-MS disease course has been reported in both children and adults.

## 5. MOG in Monophasic and Relapsing Forms of ADEM

MOG-Abs were recently detected in a subgroup of children with ADS, in particular in children with a non-MS like disease course [23,24,48].

MOG protein is expressed exclusively in the CNS and is a minor part of the myelin sheath. Autoantibodies directed against this protein are of the IgG1 subtype, induce complement-mediated cytotoxicity in vitro and transiently disrupt microtubule organization of oligodendrocytes [49,50]. The expression of serum MOG-Ab is age dependent and associated with different disease manifestations, including ADEM, ON and TM, either alone or in combination [48]. In young children, ADEM is the predominant clinical manifestation, whereas older children with MOG-Ab present with ON, myelitis or brainstem symptoms.

The clinical course of children with ADEM and MOG-Ab is primarily characterized by encephalopathy in addition to polyfocal neurological signs. Imaging of the brain and spine shows widespread involvement of different anatomical areas including the brainstem and spinal cord, often with longitudinally extensive transverse myelitis (LETM) [51]. Children whose MOG-Ab level declines to undetectable levels are less likely to have further relapses and more likely to have a favorable long-term prognosis [51].

Children with a sole monophasic ADEM or subsequent recurring demyelinating events are difficult to distinguish on clinical and radiological grounds [52]. Standard laboratory findings for children with a relapsing course, such as CSF cell count and the presence of oligoclonal bands (OCBs), are comparable to those for children with a monophasic disease course [24,48,53].

**Figure 3:**
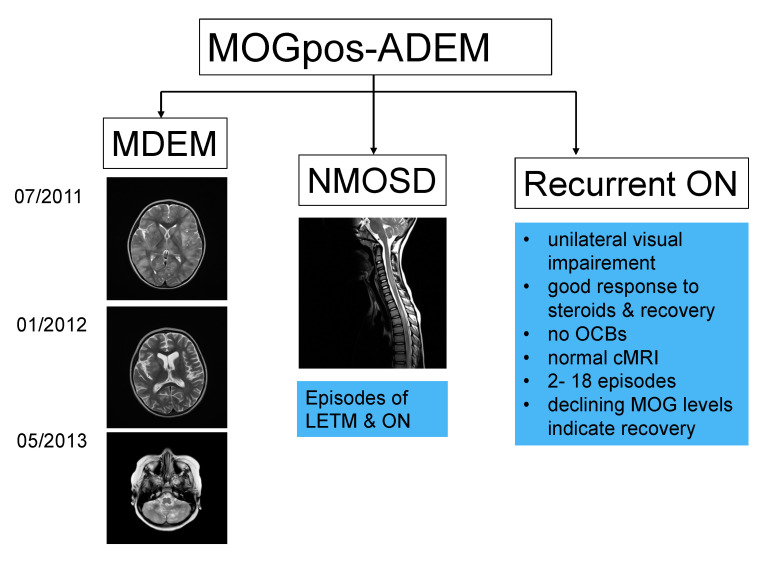
Children with acute disseminated encephalomyelitis (ADEM) and persistent myelin oligodendrocyte glycoprotein antibody (MOGpos-ADEM) are at risk of developing one of three recurrent demyelinating syndromes in addition to less well-characterized manifestations: multiphasic ADEM (MDEM), neuromyelitis optica spectrum disorder (NMOSD) generally with longitudinally extensive transverse myelitis (LETM) or recurrent optic neuritis (ON).

Recent studies focusing on the long-term outcome of children with ADS and MOG-Ab revealed that a proportion of children who initially presented with ADEM developed further demyelinating episodes such as MDEM, ADEM-ON and NMOSD in addition to clinical phenotypes with overlapping features [8,48,51,53]. Serum titers of MOG-Ab at initial presentation of children with recurrent episodes are similar to those of children with a sole monophasic event, and therefore do not seem helpful in predicting the future disease course. In addition, the time interval between the initial clinical episode and declining MOG-Ab levels can differ markedly between children with MOG-Ab-positive ADEM [48], making it difficult to predict the immediate future course once the persistence of high MOG-Ab titers is strongly associated with a recurrent non-MS disease course [48]. Figure 3 illustrates the clinical phenotypes of recurrent MOG-Ab-associated disease following ADEM.

### 5.1. MDEM

The first subgroup of children with further demyelinating episodes following ADEM were the MDEM patients. According to the last IPMSSG report, MDEM is defined as an ADEM attack followed >3 months later by a second ADEM episode without any further attacks [1,4]. Nevertheless, some patients present with more than two ADEM attacks in combination with persistent MOG-Ab [10,12,51]. The second demyelinating event generally occurs in the following 12 months, but the time interval and frequency of attacks vary considerably among patients, and can take up to four years for the second event [48]. These children develop new clinical symptoms with encephalopathy and focal neurological signs and new MRI findings characterized by hazy, large and bilateral widespread lesions [48,51]. Between MDEM episodes, children can experience events that do not fulfill MDEM criteria, presenting with severe headache in addition to widespread new lesions on cerebral MRI, or even an episode of ON without new MRI lesions [48,51]. Children with ADEM and further relapsing episodes in the context of absent serum MOG-Ab are more likely to develop an alternative diagnosis over time [48,51].

### 5.2. ADEM-ON

The second recurrent demyelinating subgroup with persistent MOG-Ab is ADEM-ON. These patients have frequent attacks of mainly unilateral inflammation of the optic nerves, ranging from one to nine episodes, occasionally in combination with further ADEM-like attacks [8,53]. Interestingly, children during the recurrent ON phase rarely show new MRI lesions. Of 17 ADEM-ON children evaluated in a recent European collaborative study, two-thirds were reported to have residual clinical symptoms including visual and cognitive impairment in addition to behavioral and bladder problems. No relapses occurred under maintenance therapy of oral prednisolone at a dosage of at least 10 mg daily [8].

The group of children with recurrent ON should be mentioned because of overlapping imaging features with ADEM-ON. Usually these children show contrast medium enhancement of the optic nerves in imaging studies only. Occasionally they exhibit MRI signs usually associated with ADEM, such as widespread supra- and infratentorial lesions without encephalopathy or additional focal neurological signs. Recurrent episodes of ON are characterized by rapid visual impairment, which usually responds to steroids, and older age at onset [54,55].

### 5.3. ADEM-NMOSD

A third group of children with MOG-Ab have further demyelinating episodes characterized by LETM and ON either simultaneously or, more often, sequentially, thus fulfilling the diagnostic criterion of AQP4-Ab-negative NMOSD. These children show a wide range of MRI findings which can be indistinguishable from children with AQP4-Ab and appear to have residual deficits more often (personal observation). As in children with MDEM or ADEM-ON, serum MOG-Ab titers remain high over time.

## 6. Pathology and Immunopathogenesis

The specific brain pathology of ADEM patients has been described. A perivascular, particularly perivenous, demyelination, giving an aspect of perivenular sleeves, has been observed with the presence of inflammatory cells, specifically macrophages, lymphocytes and microglia [56]. This perivenous demyelination is not found in other demyelinating diseases such as MS, where a more confluent demyelination is noticed. Interestingly, a multifocal pattern of activated microglia in the non-demyelinated cortex of patients with perivenous demyelination and depressed or altered consciousness has been noted. While these lesions can provide a glimpse into the types of lesions that occur and immune cells involved in ADEM, others studies tried to look for markers that may help to understand the reasons for these kinds of lesions.

Some authors looked for inflammatory markers in the CSF in demyelinating diseases and observed that in ADEM patients, inflammatory cytokines such as those related to Th1 and Th2 were increased [57]. Similarly, macrophage/microglia-related cytokines were also increased, suggesting a role of innate immunity in ADEM. In all studies, IL-6 was one of the cytokines that seemed to be regularly increased in these patients, also with a correlation with oligoclonal IgG [58] and MOG-Ab [59], which may suppose that an auto-paracrine loop between IL-6 and B/plasmocytic cells exists.

The presence of MOG-Abs is an interesting marker of ADEM in this decade and has reignited scientific interest in investigating the pathophysiology of ADEM. More than 60% of ADEM patients were positive for these antibodies, and their role remains unknown. Studies have suggested that these antibodies are able to induce complement-mediated cytotoxicity. Indeed, it has been shown that their titers are correlated with increased complement level and the extent of antibody-dependent cell-mediated cytotoxicity (ADCC) [59,60]. Interestingly, the most common MOG-Ab isotype is IgG1, which is able to fix complement and bind to Fc receptors [61]. MOG-Abs in high titers from seropositive patients were able to activate the complement cascade in vitro with complement-mediated lysis of MOG-transfected cells. Moreover, Brillot et al. showed that purified MOG-Abs from patients resulted in a loss of organization of the architecture of the microtubule cytoskeleton of oligodendroctyes without increasing cell death [50]. It was shown that purified human MOG-Abs from a patient with high titers induced strong complement activation in an organotypic brain slice [62], strongly supporting the possibility of complement-mediated activation of these antibodies. Recently, in a non-human primate (NHP) model of experimental auto-immune encephalitis (EAE) induced by recombinant human MOG, which mimicked ADEM, a similar increase in cytokines involved in innate immunity (IL-6, G-CSF, GM-CSF) was observed in the CSF. Moreover, pathological analysis of brain biopsies of children with ADEM and brain samples of a recombinant human MOG-induced EAE model showed activation of complement, confirming the possibility of complement-induced inflammation in ADEM [63].

Mechanisms of disease induction center around the loss of self-tolerance, causing a central nervous system-directed auto-reactivity that is similar across a range of neuroinflammatory conditions [64]. As the majority of ADEM cases in children are preceded by an infection, two key hypotheses have been proposed. The first is molecular mimicry: structural conformation or peptide sequences may be shared between host CNS proteins and some viral pathogens. When a viral infection occurs, a cross-reactive auto-immune reaction may be induced against the tissue expressing the mimicked protein. This was nicely shown in a transgenic mouse model, where insertion of a lymphocytic choriomeningitis virus (LCMV) antigen in murine oligodendrocytes was performed. Once these animals were inoculated intraperitoneally with the respective LCMV strain, the infection was cleared at the entry site. Following a 1- or 2-week interval, a chronic CNS inflammation occurred and was enhanced following a second infection by the same virus or an unrelated virus that could cross-activate LCMV-specific T cells [65], suggesting molecular mimicry. The second hypothesis is the post infectious theory. After the first infection by a neurotropic virus, CNS damage may occur with disruption of the blood–brain barrier (BBB). This would lead to leakage of CNS-confined auto-antigens, which would induce a breakdown of tolerance and emergence of autoreactive immune cells against CNS proteins, which could enter into the CNS through the damaged BBB [66].

## 7. Treatment and Outcome

### 7.1. Acute Phase Treatment

As there are no specific randomized trials for ADEM, treatment protocols are derived from observational studies and expert opinions. Supportive care is important, and treatment with antivirals and antibiotics is generally prescribed, as ADEM may mimic infection [26,67]. Once a diagnosis of ADEM is suspected, early treatment may contribute to a better outcome compared to historic reports [1,13,28]. First-line acute treatment generally consists of IV methylprednisolone at a dose of 30 mg/kg/day (maximum 1000 mg/day) for 3–5 days, followed by an oral prednisone taper for 4–6 weeks [1,67]. Early discontinuation of steroids (<3 weeks) can increase the risk of relapse [68]. Steroid treatment requires close monitoring of blood pressure, electrolytes and glucose and administration of gastric protection [69]. Intravenous immunoglobulin (IVIG) is prescribed as second-line treatment for steroid-unresponsive ADEM at a total dose of 2 g/kg for 2–5 days. IVIG is generally well tolerated in children [70]. The rational use of IVIG is shown in patients with recurrent or steroid-dependent demyelination [26]. An ongoing trial of early IVIG in children with encephalitis might help in reconsidering the position of IVIG in brain inflammation [71]. Plasma exchange (PLEX) with three to seven exchanges is used in refractory patients [26,32,67,72,73,74,75]. The usefulness of PLEX has been shown in trials of adults with demyelinating disorders [73] and in patients with AQP4-ab NMOSD as early therapy [74,76]. Recent pediatric studies advocate PLEX as a safe and effective rescue therapy for inflammatory CNS disorders, including ADEM [72,75]. Craniectomy has been performed in fulminant cases with increased intracranial pressure unresponsive to immunotherapy and critical care measures [69].

Recovery is expected within days after the initiation of therapy [1,26]. A plan for follow-up MRI is required to assess multiphasic disorders [67]. Although radiological frequency remains controversial, it might be reasonable to wait 3 months from the event, as radiologic findings can fluctuate in this time period.

### 7.2. Relapsing Forms

Most patients will have monophasic disease, whereas 10–36% will have another demyelinating event [52,67]. Patients diagnosed with MS or NMOSD after an ADEM episode are treated with standard disease protocols.

Therapy for recurrent MOG-Ab-associated diseases remains a challenge. In a recent European collaborative study, it was shown that disease-modifying treatments such as interferons or natalizumab, commonly used for MS patients, were not associated with clinical improvement in children with MOG-Ab-associated disease, whilst B cell-targeted treatments, particularly intravenous immunoglobulins, were associated with a reduction in relapse frequency [11]. Other reports suggested that continuous oral steroid treatment might be associated with a reduced relapse rate. Further studies are needed to address the optimal treatment and outcome in this group of children.

### 7.3. Outcome

Approximately a quarter of all children hospitalized with ADEM require admission to the intensive care unit [77]. The mortality rate was reported as 1–3% [1,77]. Even though there is no uniform evaluation among different ADEM studies, full recovery with normal neurological examination is reported for most patients (50–80%) [67,70]. Long-term motor deficits, visual problems and seizures are rare [14]. Distinct cognitive problems are reported in up to 56% of patients [15,67,69]. Recent pediatric MOG-Ab studies found that poor cognitive outcome was associated with the ADEM or ADEM-ON phenotype [11,51,78] at a higher frequency compared to adult studies [8]. Academic disorders as an indirect measure of poor cognition were also associated with ADEM and younger age at onset and deep gray matter lesions on MRI scans [79]. A recent evaluation of children with acquired demyelination demonstrated reduced brain volume and reduced expected brain growth even in the absence of chronicity, especially in 18 ADEM patients. It is postulated that a higher volume of white matter lesions in ADEM patients and their younger age at onset might be responsible for the lack of expected white matter growth in these patients [80].

## 8. Future Directions

With the advance of MOG-Ab and the renewed interest in understanding neurobiology, interesting work is arising [13]. Evidence of MOG-Ab pathogenicity and persistence in children with both monophasic and relapsing forms of ADEM is still evolving [81]. As for the differentiation, management and prognosis of other CNS demyelinating disorders, antibody positivity is essential [9]. The role of MOG-Ab and the serostatus of other antibodies must be addressed in future ADEM diagnostic criteria [6,10]. Newer therapies are emerging with an understanding of the pathobiological mechanisms, genetics and biomarkers of different neuroimmune disorders [63]. Changes in the management of relapsing ADEM cases tend to be well directed by antibody positivity. Consistent data on treatment in the acute phase are still lacking. Long-term outcomes from collaborative multicenter studies are required in this new era, including more sensitive tools to especially evaluate cognitive outcomes [78].

## Figures and Tables

**Table 1:** Criteria for acute disseminated encephalomyelitis (ADEM) and relapsing disorders following ADEM.

ADEM	Single polyfocal clinical CNS event with presumed inflammatory cause Encephalopathy that cannot be explained by fever MRI typically shows diffuse, poorly demarcated, large >1–2 cm lesions predominantly involving cerebral white matter; T1 hypointense white matter lesions are rare; deep gray matter lesions (e.g., thalamus or basal ganglia) can be present No new symptoms, signs or MRI findings after three months of initial ADEM
Multiphasic ADEM	New event of ADEM three months or more after initial event that can be associated with new or re-emergence of prior clinical and MRI findings
ADEM-ON	At least one subsequent attack of optic neuritis, without encephalopathy, at least three months after initial ADEM
ADEM-MS	ADEM followed three months later by a non-encephalopathic clinical event with new lesions on brain MRI consistent with MS
ADEM-NMOSD	ADEM followed three months later by ON, myelitis or area postrema syndrome, fulfilling NMOSD diagnostic criteria

ADEM, acute disseminated encephalomyelitis; CNS, central nervous system; ON, optic neuritis; MRI, magnetic resonance imaging; MS, multiple sclerosis; NMOSD, neuromyelitis optica spectrum disorder. Adapted from Pohl et al. [1].

## References

[1] Pohl D., Alper G., Haren K.V., Kornberg A.J., Lucchinetti C.F., Tenembaum S., Belman A.L. (2016). Acute disseminated encephalomyelitis. Updates on an inflammatory CNS syndrome. Neurology.

[2] Belman A.L., Hertz D., Hanefeld F., Chabas D., Waubant E.L. (2011). Introduction: Historical perspective of pediatric multiple sclerosis and related disorders. Demyelinating Disorders of the Central Nervous System in Childhood.

[3] Krupp L.B., Banwell B., Tenembaum S. (2007). Consensus definitions proposed for pediatric multiple sclerosis and related disorders. Neurology.

[4] Krupp L.B., Tardieu M., Amato M.P., Banwell B., Chitnis T., Dale R.C., Ghezzi A., Hintzen R., Kornberg A., Pohl D. (2013). International pediatric multiple sclerosis study group criteria for pediatric multiple sclerosis and immune-mediated central nervous system demyelinating disorders: Revisions to the 2007 definitions. Mult. Scler..

[5] Granerod J., Davies N.W.S., Mukonoweshuro W., Mehta A., Das K., Lim M., Solomong T., Biswas S., Rosella L., Brown D.W.G. (2016). Neuroimaging in encephalitis: Analysis of imaging findings and interobserver agreement. Clin. Radiol..

[6] Hardy T.A. (2018). How should we diagnose acute disseminated encephalomyelitis?. Dev. Med. Child Neurol..

[7] Boesen M.S., Blinkenberg M., Koch-Henriksen N., Thygesen L.C., Uldall P.V., Magyari M., Born A.P. (2018). Implications of the international paediatric multiple sclerosis study group consensus criteria for paediatric acute disseminated encephalomyelitis: A nationwide validation study. Dev. Med. Child Neurol..

[8] Wong Y.Y.M., Hacohen Y., Armangue T., Wassmer E., Verhelst H., Hemingway C., van Pelt E.D., Catsman-Berrevoets C.E., Hintzen P.Q., Deiva K. (2018). Paediatric acute disseminated encephalomyelitis followed by optic neuritis: Disease course, treatment response and outcome. Eur. J. Neurol..

[9] Hacohen Y., Mankad K., Chong W.K., Barkhof F., Vincent A., Lim M., Wassmer E., Ciccarelli O., Hemingway C. (2017). Diagnostic algorithm for relapsing acquired demyelinating syndromes in children. Neurology.

[10] Armangue T., Olivé-Cirera G., Martínez-Hernandez E., Sepulveda M., Ruiz-Garcia R., Muñoz-Batista M., Ariño H., González-Álvarez V., Felipe-Rucián A., Martínez-González M.J. (2020). Associations of paediatric demyelinating and encephalitic syndromes with myelin oligodendrocyte glycoprotein antibodies: A multicentre observational study. Lancet Neurol..

[11] Hacohen Y., Wong Y.Y., Lechner C., Jurynczyk M., Wright S., Konuskan B., Kalser J., Poulat A.L., Maurey H., Ganelin-Cohen E. (2018). Disease course and treatment responses in children with relapsing myelin oligodendrocyte glycoprotein antibody–associated disease. JAMA Neurol..

[12] Waters P., Fadda G., Woodhall M., O’Mahony J., Brown R.A., Castro D.A., Longoni G., Irani S.R., Sun B., Yeh E.A. (2020). Serial anti–myelin oligodendrocyte glycoprotein antibody analyses and outcomes in children with demyelinating syndromes. JAMA Neurol..

[13] Wells E., Hacohen Y., Waldman A., Tillema J.M., Soldatos A., Ances B., Benseler S., Bielekova B., Dale R.C., Dalmau J. (2018). Neuroimmune disorders of the central nervous system in children in the molecular era. Nat. Rev. Neurol..

[14] Tenembaum S., Chamoles N., Fejerman N. (2002). Acute disseminated encephalomyelitis. A long-term follow-up study of 84 pediatric patients. Neurology.

[15] Hahn C.D., Miles B.S., MacGregor D.L., Blaser S.I., Banwell B.L., Hetherington C.R. (2003). Neurocognitive outcome after acute disseminated encephalomyelitis. Pediatric Neurol..

[16] Neuteboom R., Wilbur C., Van Pelt D., Rodriguez M., Yeh A. (2017). The spectrum of inflammatory acquired demyelinating syndromes in children. Semin. Pediatric Neurol..

[17] Banwell B., Kennedy J., Sadovnick D., Arnold D.L., Magalhaes S., Wambera K., Connolly M.B., Yager J., Mah J.K., Shah N. (2009). Incidence of acquired demyelination of the CNS in Canadian children. Neurology.

[18] Hintzen R.Q., Dale R.C., Neuteboom R.F., Mar S., Banwell B. (2016). Pediatric acquired CNS demyelinating syndromes. Features associated with multiple sclerosis. Neurology.

[19] Ketelslegers I.A., Catsman-Berrevoets C.E., Neuteboom R.F., Boon M., van Dijk K.G., Eikelenboom M.J., Gooskens R.H., Niks E.H., Overweg-Plandsoen W.C., Peeters E.A. (2012). Incidence of acquired demyelinating syndromes of the CNS in Dutch children: A nationwide study. J. Neurol..

[20] Mikaeloff Y., Suissa S., Vallée L., Lubetzki C., Ponsot G., Confavreux C., Tardieu M. (2004). First episode of acute CNS inflammatory demyelination in childhood: Prognostic factors for multiple sclerosis and disability. J. Pediatric.

[21] Neuteboom R.F., Boon M., Catsman Berrevoets C.E., Vles J.S., Gooskens R.H., Stroink H., Vermeulen R.J., Rotteveel J.J., Ketelslegers I.A., Peeters E. (2008). Prognostic factors after a first attack of inflammatory CNS demyelination in children. Neurology.

[22] Tardieu M., Mikaeloff Y. (2004). What is acute disseminated encephalomyelitis (ADEM)?. Eur. J. Paediatr. Neurol..

[23] Ketelslegers I.A., Pelt D.E.V., Bryde S., Neuteboom R.F., Catsman-Berrevoets C.E., Hamann D., Hintzen R.Q. (2015). Anti-MOG antibodies plead against MS diagnosis in an acquired demyelinating syndromes cohort. Mult. Scler. J..

[24] Hacohen Y., Absoud M., Deiva K., Hemingway C., Nytrova P., Woodhall M., Palace J., Wassmer E., Tardieu M., Vincent A. (2015). Myelin oligodendrocyte glycoprotein antibodies are associated with a non-MS course in children. Neurol. Neuroimmunol. Neuroinflamm..

[25] Wingerchuk D.M., Banwell B., Bennett J.L., Cabre P., Carroll W., Chitnis T., de Seze J., Fujihara K., Greenberg B., Jacob A. (2015). International consensus diagnostic criteria for neuromyelitis optica spectrum disorders. Neurology.

[26] Tenembaum S.N. (2013). Acute disseminated encephalomyelitis. Pediatric neurology Part II. Handbook of Clinical Neurology.

[27] Parrish J.B., Yeh E.A., Ahmad S.I. (2012). Acute disseminated encephalomyelitis. Neurodegenerative Diseases.

[28] Berzero G., Cortese A., Ravaglia S., Marchioni E. (2015). Diagnosis and therapy of acute disseminated encephalomyelitis and its variants. Expert Rev. Neurother..

[29] de Mol C.L., Wong Y.Y.M., van Pelt E.D., Ketelslegers I.A., Bakker D.P., Boon M., Braun K.P.J., van Dijk K.G.J., Eikelenboom M.J., Engelen M. (2018). Incidence and outcome of acquired demyelinating syndromes in Dutch children: Update of a nationwide and prospective study. J. Neurol..

[30] Absoud M., Lim M.J., Chong W.K., De Goede C.G., Foster K., Gunny R., Hemingway C., Jardine P.E., Kneen R., Likeman M. (2013). Paediatric acquired demyelinating syndromes: Incidence, clinical and magnetic resonance imaging features. Mult. Scler..

[31] Pellegrino P., Radice S., Clementi E. (2014). Geoepidemiology of acute disseminated encephalomyelitis. Epidemiology.

[32] Koelman D.L., Mateen F.J. (2015). Acute disseminated encephalomyelitis: Current controversies in diagnosis and outcome. J. Neurol..

[33] Karussis D., Petrou P. (2014). The spectrum of post-vaccination inflammatory CNS demyelinating syndromes. Autoimmun. Rev..

[34] Langer-Gould A., Qian L., Tartof S.Y., Brara S.M., Jacobsen S.J., Beaber B.E., Sy L.S., Chao C., Hechter R., Tseng H.F. (2014). Vaccines and the risk of multiple sclerosis and other central nervous system demyelinating diseases. JAMA Neurol..

[35] Koelman D.L.H., Chahin C., Mar S.S., Venkatesan A., Hoganson G.M., Yeshokumar A.K., Barreras P., Majmudar B., Klein J.P., Chitnis T. (2016). Acute disseminated encephalomyelitis in 228 patients. A retrospective, multicenter US study. Neurology.

[36] Dundar N.O., Anlar B., Guven A., Serdaroglu A., Yarar C. (2010). Relapsing acute disseminated encephalomyelitis in children: Further evaluation of the diagnosis. J. Child Neurol..

[37] Tenembaum S., Chitnis T., Ness J., Hahn J.S. (2007). Acute disseminated encephalomyelitis. Neurology.

[38] Ketelslegers I.A., Visser I.E., Neuteboom R.F., Boon M., Catsman-Berrevoets C.E., Hintzen R.Q. (2011). Disease course and outcome of acute disseminated encephalomyelitis is more severe in adults than in children. Mult. Scler..

[39] Werner K.M., Dosh M.P. (2018). Atypical ADEM and cardiogenic shock in a 14-year-old female. Pediatrics.

[40] Lademann H., Bertsche A., Petzold A., Zack F., Buttner A., Dabritz J., Hauenstein C., Bahn E., Spang C., Reuter D. (2020). Acute disseminated encephalomyelitis with seizures and myocarditis: A fatal triad. Medicina.

[41] Dale R.C., Sousa C., Chong W.K., Cox T.C.S., Harding B., Neville B.G.R. (2000). Acute disseminated encephalomyelitis, multiphasic disseminated encephalomyelitis and multiple sclerosis in children. Brain.

[42] Wong Y.Y.M., van Pelt E.D., Ketelslegers I.A., Catsman-Berrevoets C.E., Hintzen R.Q., Neuteboom R.F. (2017). Evolution of MRI abnormalities in paediatric acute disseminated encephalomyelitis. Eur. J. Paediatr. Neurol..

[43] Callen D.J., Shroff M.M., Branson H.M., Li D.K., Lotze T., Stephens D., Banwell B.L. (2009). Role of MRI in the differentiation of ADEM from MS in children. Neurology.

[44] Ketelslegers I.A., Neuteboom R.F., Boon M., Catsman-Berrevoets C.E., Hintzen R.Q. (2010). A comparison of MRI criteria for diagnosing pediatric ADEM and MS. Neurology.

[45] Pavone P., Pettoello-Mantovano M., Le Pira A., Giardino I., Pulvirenti A., Giugno R., Parano E., Polizzi A., Distefano A., Ferro A. (2010). Acute disseminated encephalomyelitis: A long-term prospective study and meta-analysis. Neuropediatrics.

[46] Kahlmann V., Roodbol J., van Leeuwen N., Ramakers C.R.B., van Pelt D., Neuteboom R.F., Catsman-Berrevoets C.E., de Wit M.C.Y., Jacobs B.C. (2017). Validated age-specific reference values for CSF total protein levels in children. Eur. J. Paediatr. Neurol..

[47] Rostasy K., Bajer-Kornek B., Venkateswaran S., Hemingway C., Tardieu M. (2016). Differential diagnosis and evaluation in pediatric inflammatory demyelinating disorders. Neurology.

[48] Hennes E., Baumann M., Schanda K., Anlar B., Bajer-Kornek B., Blaschek A., Brantner-Inthaler S., Diepold K., Eisenkölbl A., Gotwald T. (2017). Prognostic relevance of MOG antibodies in children with an acquired demyelinating syndrome. Neurology.

[49] Mader S., Gredler V., Schanda K., Rostasy K., Dujmovic I., Pfaller K., Lutterotti A., Jarius S., Di Pauli F., Kuenz B. (2011). Complement activating antibodies to myelin oligodendrocyte glycoprotein in neuromyelitis optica and related disorders. J. Neuroinflamm..

[50] Dale R.C., Tantsis E.M., Merheb V., Kumaran R.Y., Sinmaz N., Pathmanandavel K., Ramanathan S., Booth D.R., Wienholt L.A., Prelog K. (2014). Antibodies to MOG have a demyelination phenotype and affect oligodendrocyte cytoskeleton. Neurol. Neuroimmunol. Neuroinflamm..

[51] Baumann M., Hennes E., Schanda K., Karenfort M., Kornek B., Seidl R., Diepold K., Lauffer H., Marquardt I., Strautmanis J. (2016). Children with multiphasic disseminated encephalomyelitis and antibodies to the myelin oligodendrocyte glycoprotein (MOG): Extending the spectrum of MOG antibody positive diseases. Mult. Scler..

[52] Kariyawasam S., Singh R.R., Gadian J., Lumsden D.E., Lin J., Siddiqui A., Hacohen Y., Absoud M., Lim M. (2015). Clinical and radiological features of recurrent demyelination following acute disseminated encephalomyelitis (ADEM). Mult. Scler. Relat. Disord..

[53] Huppke P., Rostasy K., Karenfort M., Huppke B., Seidl R., Leiz S., Reindl M., Gartner J. (2013). Acute disseminated encephalomyelitis followed by recurrent or monophasic optic neuritis in pediatric patients. Mult. Scler..

[54] Rostasy K., Mader S., Schanda K., Huppke P., Gartner J., Kraus V., Karenfort M., Tibussek D., Blaschek A., Bajer-Kornek B. (2012). Anti-myelin oligodendrocyte glycoprotein antibodies in pediatric patients with optic neuritis. Arch. Neurol..

[55] Ramanathan S., Prelog K., Barnes E.H., Tantsis E.M., Reddel S.W., Henderson A.P.D., Vucic S., Gorman M.P., Benson L.A., Alper G. (2016). Radiological differentiation of optic neuritis with myelin oligodendrocyte glycoprotein antibodies, aquaporin-4 antibodies, and multiple sclerosis. Mult. Scler..

[56] Young N.P., Weinshenker B.G., Parisi J.E., Scheithauer B., Giannini C., Roemer S.F., Thomsen K.M., Mandrekar J.N., Erickson B.J., Lucchinetti C.F. (2010). Perivenous demyelination: Association with clinically defined acute disseminated encephalomyelitis and comparison with pathologically confirmed multiple sclerosis. Brain.

[57] Ishizu T., Minohara M., Ichiyama T., Kira R., Tanaka M., Osoegawa M., Hara T., Furukawa S., Kira J. (2006). CSF cytokine and chemokine profiles in acute disseminated encephalomyelitis. J. Neuroimmunol..

[58] Dale R.C., Morovat A. (2003). Interleukin-6 and oligoclonal igg synthesis in children with acute disseminated encephalomyelitis. Neuropediatrics.

[59] Horellou P., Wang M., Keo V., Chretien P., Serguera C., Waters P., Deiva K. (2015). Increased interleukin-6 correlates with myelin oligodendrocyte glycoprotein antibodies in pediatric monophasic demyelinating diseases and multiple sclerosis. J. Neuroimmunol..

[60] Brilot F., Dale R.C., Selter R.C., Grummel V., Kalluri S.R., Aslam M., Busch V., Zhou D., Cepok S., Hemmer B. (2009). Antibodies to native myelin oligodendrocyte glycoprotein in children with inflammatory demyelinating central nervous system disease. Ann. Neurol..

[61] McLaughlin K.A., Chitnis T., Newcombe J., Franz B., Kennedy J., McArdel S., Kuhle J., Kappos L., Rostasy K., Pohl D. (2009). Age-dependent B cell autoimmunity to a myelin surface antigen in pediatric multiple sclerosis. J. Immunol..

[62] Peschl P., Schanda K., Zeka B., Given K., Bohm D., Ruprecht K., Saiz A., Lutterotti A., Rostasy K., Hoftberger R. (2017). Human antibodies against the myelin oligodendrocyte glycoprotein can cause complement-dependent demyelination. J. Neuroinflamm..

[63] Fovet C.M., Stimmer L., Contreras V., Horellou P., Hubert A., Seddiki N., Chapon C., Tricot S., Leroy C., Flament J. (2019). Intradermal vaccination prevents anti-MOG autoimmune encephalomyelitis in macaques. EBioMedicine.

[64] Bhat R., Steinman L. (2009). Innate and adaptive autoimmunity directed to the central nervous system. Neuron.

[65] Evans C.F., Horwitz M.S., Hobbs M.V., Oldstone M.B.A. (1996). Viral infection of transgenic mice expressing a viral protein in oligodendrocytes leads to chronic central nervous system autoimmune disease. J. Exp. Med..

[66] Mengea T., Kieseiera B.C., Nesslera S., Hemmera B., Hartunga H., Stüve O. (2007). Acute disseminated encephalomyelitis: An acute hit against the brain. Curr. Opin. Neurol..

[67] Cole J., Evans E., Mwangi M., Mar S. (2019). Acute disseminated encephalomyelitis in children: An updated review based on current diagnostic criteria. Pediatr. Neurol..

[68] Anlar B., Basaran C., Kose G., Guven A., Haspolat S., Yakut A., Serdaroglu A., Senbil N., Tan H., Karaagaoglu E. (2003). Acute disseminated encephalomyelitis in children: Outcome and prognosis. Neuropediatrics.

[69] Pohl D., Tenembaum S. (2012). Treatment of acute disseminated encephalomyelitis. Curr. Treat Options Neurol..

[70] Chitnis T. (2013). Pediatric demyelinating diseases. Contin. (Minneap Minn).

[71] Iro M.A., Sadarangani M., Absoud M., Chong W.K., Clark C.A., Easton A., Gray V., Kneen R., Lim M., Pike M. (2016). ImmunoglobuliN in the treatment of encephalitis (IgNiTE): Protocol for a multicentre randomised controlled trial. BMJ Open.

[72] Eyre M., Hacohen Y., Barton C., Hemingway C., Lim M. (2018). Therapeutic plasma exchange in paediatric neurology: A critical review and proposed treatment algorithm. Dev. Med. Child Neurol..

[73] Weinshenker B.G., O’Brien P.C., Petterson T.M., Noseworthy J.H., Lucchinetti C.F., Dodick D.W., Pineda A.A., Stevens L.N., Rodriguez M. (1999). A randomized trial of plasma exchange in acute central nervous system inflammatory demyelinating disease. Ann. Neurol..

[74] Abboud H., Petrak A., Mealy M., Sasidharan S., Siddique L., Levy M. (2016). Treatment of acute relapses in neuromyelitis optica: Steroids alone versus steroids plus plasma exchange. Mult. Scler..

[75] Savransky A., Rubstein A., Rios M.H., Vergel S.L., Velasquez M.C., Sierra S.P., Marcarian G., Alba R., Pugliese A.M., Tenembaum S. (2019). Prognostic indicators of improvement with therapeutic plasma exchange in pediatric demyelination. Neurology.

[76] Kim S.H., Kim W., Huh S.Y., Lee K.Y., Jung I.J., Kim H.J. (2013). Clinical efficacy of plasmapheresis in patients with neuromyelitis optica spectrum disorder and effects on circulating anti-aquaporin-4 antibody levels. J. Clin. Neurol..

[77] Absoud M., Parslow R.C., Wassmer E., Hemingway C., Duncan H.P., Cummins C., Lim M.J. (2010). Severe acute disseminated encephalomyelitis: A paediatric intensive care population-based study. Mult. Scler..

[78] Zhou J., Lu X., Zhang Y., Ji T., Jin Y., Xu M., Bao X., Zhang Y., Xiong H., Chang X. (2019). Follow-up study on Chinese children with relapsing MOG-IgG-associated central nervous system demyelination. Mult. Scler. Relat. Disord..

[79] Deiva K., Cobo-Calvo A., Maurey H., De Chalus A., Yazbeck E., Husson B., Vukusic S., Serguerra C., Horellou P., Marignier R. (2020). Risk factors for academic difficulties in children with myelin oligodendrocyte glycoprotein antibody-associated acute demyelinating syndromes. Dev. Med. Child Neurol..

[80] Aubert-Broche B., Weier K., Longoni G., Fonov V.S., Bar-Or A., Marrie R.A., Yeh E.A., Narayanan S., Arnold D.L., Verhey L.H. (2017). Monophasic demyelination reduces brain growth in children. Neurology.

[81] Spadaro M., Winklmeier S., Beltran E., Macrini C., Hoftberger R., Schuh E., Thaler F.S., Gerdes L.A., Laurent S., Gerhards R. (2018). Pathogenicity of human antibodies against myelin oligodendrocyte glycoprotein. Ann. Neurol..

